# Exercise training-induced changes in immunometabolic markers in youth badminton athletes

**DOI:** 10.1038/s41598-022-19591-w

**Published:** 2022-09-15

**Authors:** Fabrício Eduardo Rossi, Alberto Jimenez Maldonado, Jason Michael Cholewa, Sergio Luiz Galan Ribeiro, Clara Andressa de Araújo Barros, Caique Figueiredo, Thomas Reichel, Karsten Krüger, Fábio Santos Lira, Luciele Guerra Minuzzi

**Affiliations:** 1 Immunometabolism of Skeletal Muscle and Exercise Research Group, Department of Physical Education, Federal University of Piaui (UFPI), Teresina, PI Brazil; 2 Graduate Program in Science and Health, Department of Physical Education, Federal University of Piaui (UFPI), “Ministro Petrônio Portella” Campus, Teresina, PI 64049-550 Brazil; 3 Facultad de Deportes Campus Ensenada, Universidad Autónoma de Baja California Mx, Mexicali, Mexico; 4 Department of Exercise Physiology, University of Lynchburg, Lynchburg, VA USA; 5 Exercise and Immunometabolism Research Group, Postgraduation Program in Movement Sciences, Department of Physical Education, Universidade Estadual Paulista (UNESP), Presidente Prudente, Brazil; 6 Department of Exercise Physiology and Sports Therapy, Institute of Sport Science, Justus-Liebig-University Gießen, 35394 Gießen, Germany; 7 Research Center for Sport and Physical Activity, Faculty of Sports Science and Physical Education, University of Coimbra, Coimbra, Portugal

**Keywords:** Cytokines, Metabolism

## Abstract

The aim of this study was to investigate the metabolic and inflammatory fluctuations in two seasonal phases of badminton training, and the ability of youth badminton athletes to respond to an inflammatory challenge given by acute exercise on these markers. Thirteen youth badminton athletes who participated in national and international competitions were recruited. Metabolic and cytokine profile were measured at rest and in response to a maximal exercise intermittent test, in the pre- and final phases of a badminton annual season. At rest, glucose (–7.58 mg/dL; p = 0.045) and HDL-cholesterol (HDL-c) (–26.87 mg/dL; p < 0.0001) decreased at final-season. The variation of HDL-c in response to a maximal exercise test increased at final-season in comparison to pre-season (+ 10.20 mg/dL p = 0.042). Similarly, delta changes of IL-10 (+ 3.41 pg/ml; p = 0.047) and IL-1Ra (+ 141.3 pg/ml; p = 0.031) were greater at final-season. In addition, a significantly greater variation of the anti-inflammatory IL-10/IL-17 ratio was observed at final-season (+ 0.37; p = 0.010). In conclusion, our results showed a major responsivity of IL-10 and IL-1Ra to a maximal exercise even at the end of an entire season. The major responsivity of these cytokines at this time point suggests a more effective acute inflammatory response in youth badminton athletes. Therefore, the results of this study may be applied by coaches, trainers and sport nutritionist for proper training management.

## Introduction

Badminton is an Olympic racket sport composed of short- and high-intensity actions with fast and explosive movements^1^. During the annual season, badminton athletes are regularly exposed to exhaustive training loads and insufficient recovery periods. This in turn can lead to a loss in performance and increased risks of injury, overreaching and potential overtraining syndromes^2^. For example, Santos et al. showed that the period of the season directly impacts some physiological and psychological factors, in which the decrease of serum BDNF levels, sleep time and carbohydrate intake were observed at the end of the season^3^. These data raised the possibility that changes in immunometabolic responses could be also observed throughout an entire competitive season in youth badminton athletes.

Both acute exercise and regular exercise training stress energy metabolism are accompanied by an exercise-induced immune response as evidenced by changes in circulating plasma cytokines^4^. While the general immunometabolic response to exercise has been well studied, the metabolic and immune responses specific to racket sports players is poorly investigated. In a previous analysis, inflammatory and muscle damage markers were increased in badminton players after a habitual training microcycle^5^. It was also suggested that their inflammatory profile was altered in response to different recovery-stress states^5^. Furthermore, large interindividual differences^5,6^, testing^7^ and opposite outcomes in inflammatory markers between badminton athletes has been also observed^5^. Thus, the lack of reference data for inflammatory markers in badminton athletes does not allow an adequate screening of muscle recovery throughout the annual competitive calendar^6^.

Furthermore, ensuring effective training involves a complex process of controlling the imposed training load and monitoring the immunometabolic response to the stimulus across the competitive season. Hence, the regular measurement of metabolic and inflammatory markers, such as cytokines, may be used as an indicator of acute and chronic training stress associated with training adaptation or, in the worst-case scenario, the development of overtraining. Although basal concentrations of serum cytokines show interindividual variability, being produced in a transient nature in response to exercise, regular monitoring for chronic changes in resting concentrations provide insight into the interaction between training load, recovery, and readiness of the athlete^8^.

In addition, the inflammatory stimulus given by acute exercise trigger anti-inflammatory responses after one bout of exercise^9^, where exercise-induced muscle damage and energy imbalance increase the synthesis and subsequent release of Interleukin (IL)-6 from contracting muscles. IL-6 is a cytokine featuring pleiotropic responses, inducing both pro- and anti-inflammatory responses^10^. It has been reported that the increase in IL-6 in the bloodstream stimulates the production of antagonistic receptor of IL-1 (IL-1Ra), IL-10 and tumour necrosis factor alpha (TNF-α)^10–12^. Chronically, these transient changes in immune and inflammatory markers evoked by a single bout of exercise may be linked to the improvement of the athletic performance and health benefits of regular exercise in badminton players. Therefore, to understand the influence of a competitive season on markers of stress and recovery, measuring the metabolic and inflammatory markers in youth badminton athletes is necessary to provide important information for coaches about athlete responses to different training loads and competitions in badminton athletes.

Compared to previous studies, which mostly analysed resting values after an entire tournament season^6^ or short-term responses after acute bouts of exercise^5,13^, in the present study we are primarily interested in detecting whether potential immunometabolic responses can be consistently observed in two contrasting season phases (early and late) in youth badminton athletes. Mostly important, whether badminton training modifies the acute cytokine response to an inflammatory stimulus given by acute exercise during different periods of the competitive season. Our hypothesis is that these athletes would present an increase in metabolic and inflammatory markers at the end of season. Thus, is expected that the immune-inflammatory and metabolic responses would change with training load, with badminton athletes showing an elevation in metabolic and inflammatory markers during the annual season as a “residual effect” regarding the demands on the sport accrued through the annual season.

Therefore, the aim of the study was to investigate the metabolic and inflammatory fluctuations in two seasonal phases using resting values in youth badminton athletes. Furthermore, the ability of the athletes to respond to an inflammatory challenge given by maximal acute aerobic test on these parameters were compared between the two seasonal phases and tested for differences.

## Methods

### Participants and training routine

Thirteen youth badminton athletes (5 males: age = 16.8 ± 1.8 years and 8 females: age = 16.3 ± 1.8 years) who participated in national and international competitions were recruited. Exclusion criteria included the use of medication or presence of metabolic disturbances that might affect blood analysis. The study was carried out between pre-season (January) and at final-season (November) of annual badminton training before the pandemic of Covid-19. Athletes trained six times per week, in a total of approximately 24 h, and their routine included general strength and endurance training in addition to basic technique work at pre-season. At final-season, athletes trained six times per week (18 h/week), with their routine involving a higher volume of technical and tactical skills as well as sport specific capacity exercises﻿.﻿

### Blood sampling and physical fitness assessment

The procedures of blood sampling and the physical fitness assessment were performed as previously reported^3^. Briefly, food intake was recorded for three non-consecutive days and the database of Brazilian food composition table (TACO) was used to calculate the dietary intake of all participants. Sleep periods were also identified using the Actilife software from Actigraph (Pensacola, FL, USA) (version 6.13.0.). Habitual physical activity and training routine were monitored using Actigraph GT3  accelerometers (ActiGraph, Ft. Walton Beach, United States).

At the day of the experimental test, a regular breakfast was provided to all participants before the aerobic exercise test (1h30min). For standardization, energy intake of breakfast on the testing day was fixed at 25% of the estimated daily energy need of each participant, consisting of 15–20% protein, 50–60% carbohydrates and 25–30% fat^14^. Resting blood samples (20 ml) were collected in a non-fasted state, with venous blood obtained from the antecubital vein into tubes containing EDTA (Gel BD SST^®^ II Advance^®^). The Yo-Yo Endurance Test 2, a high-intensity and intermittent exercise test, was applied since it measures the capacity to perform intense intermittent exercise with a large anaerobic component in combination with significant aerobic contribution^15^. Thus, Yo-Yo test simulates the badminton training as well as evokes an inflammatory challenge, making it possible to investigate how young badminton athletes respond to the inflammatory stimulus. The maximal oxygen consumption (VO_2max_) was calculated using the equation proposed by Léger et al.: 24.4 + 6 × [final velocity (km h^−1^)] for athletes aged ≥ 18 years or 31.025 + (3.238 × final velocity)—(3.248 × age) + 0.1536 × (final velocity × age) for athletes aged < 18 years^16^. Another blood sample was obtained following the Yo-Yo test, and the tubes were centrifuged (Becton Dickinson, BD, Juiz de Fora, MG, Brazil) at 1308*g* for 15 min at 4 °C, and serum samples were stored at −40 °C until analysis.

### Metabolic and inflammatory profile

Metabolic and cytokine profile were analysed before and immediately after the aerobic exercise test during both season phases. Triglycerides (TAG), total cholesterol (TC) and high-density lipoprotein cholesterol (HDL-c) were analysed with commercial colorimetric kits (Labtest, Brazil), and Non-HDL cholesterol was calculated by subtracting HDL-c concentration from TC. Glucose content was obtained with a colorimetric kit (Labtest^®^, Brazil). The concentrations of IL-6, IL-10, TNF-α were acquired by the enzyme-linked immunosorbent assay (ELISA) with commercial kits (Quantikine R&D System, Minneapolis, USA) with ranges between 3.13–100 pg/mL, 7.8–500 pg/mL and 15.6–1000 pg/mL, respectively. Adiponectin, leptin, IL-1Ra, IL-17, interferon-gamma (IFN-γ), monocyte chemoattractant protein-1 (MCP-1) and macrophage inflammatory protein-1 alpha (MIP-1α) concentrations were analysed using a Duoset^®^ ELISA assay (R&D System, Minneapolis, MN, USA), with ranges between 62.5–4000 pg/mL, 31.2–2000, 39.1–2.500 pg/mL, 15.6–1000 pg/mL, 15.6–1000 pg/mL, 15.6–1000 pg/mL and 7.81–500 pg/mL, respectively. All the assays were performed following the manufacturer´s guidelines. The intra- and inter-assay variations (%) and the sensitivity of the enzymatic kits are presented in the Supplementary Table 1.

The relationships between adiponectin and leptin, TNF-α and IL-10, and IL-17A and IL-10 are presented as metabolic (Adip/Lep), anti-inflammatory (IL-10/TNF-α; IL-10/IL-17), and inflammatory (TNF-α/IL-10, IL-17/IL-10) ratios.

### Statistical analyses

Data distribution was tested by the Shapiro–Wilk test. Paired student t-test (parametric) or Wilcoxon test (nonparametric) were used to verify differences between groups for resting values. Data are presented in values of mean and standard deviation or median and interquartile range, respectively, with the Confidence interval (CI) set at 95%. The absolute (delta) and relative (fold) changes were calculated at pre-season and final-season (delta = post-exercise minus pre-exercise; fold = post-exercise/pre-exercise) and the comparison between delta was conducted. The Pearson correlation was calculated between delta cytokine and delta VO_2max_. The SAS^®^ 26 and GraphPad Prism 9.1.2 software packages were used to analyse all data. The level of significance was set at p ≤ 0.05.

### Ethics approval

Subjects signed the free and informed consent form as their parents or guardians signed the informed and clarified consent form, both previously approved by the Ethics and Research Committee of the Federal University of Piaui (Protocol number: 2.552.506). This study was performed according to the 2013 Revision of the Declaration of Helsinki.

## Results

### Metabolic and inflammatory profile of the athletes at rest

**Figure 1:**
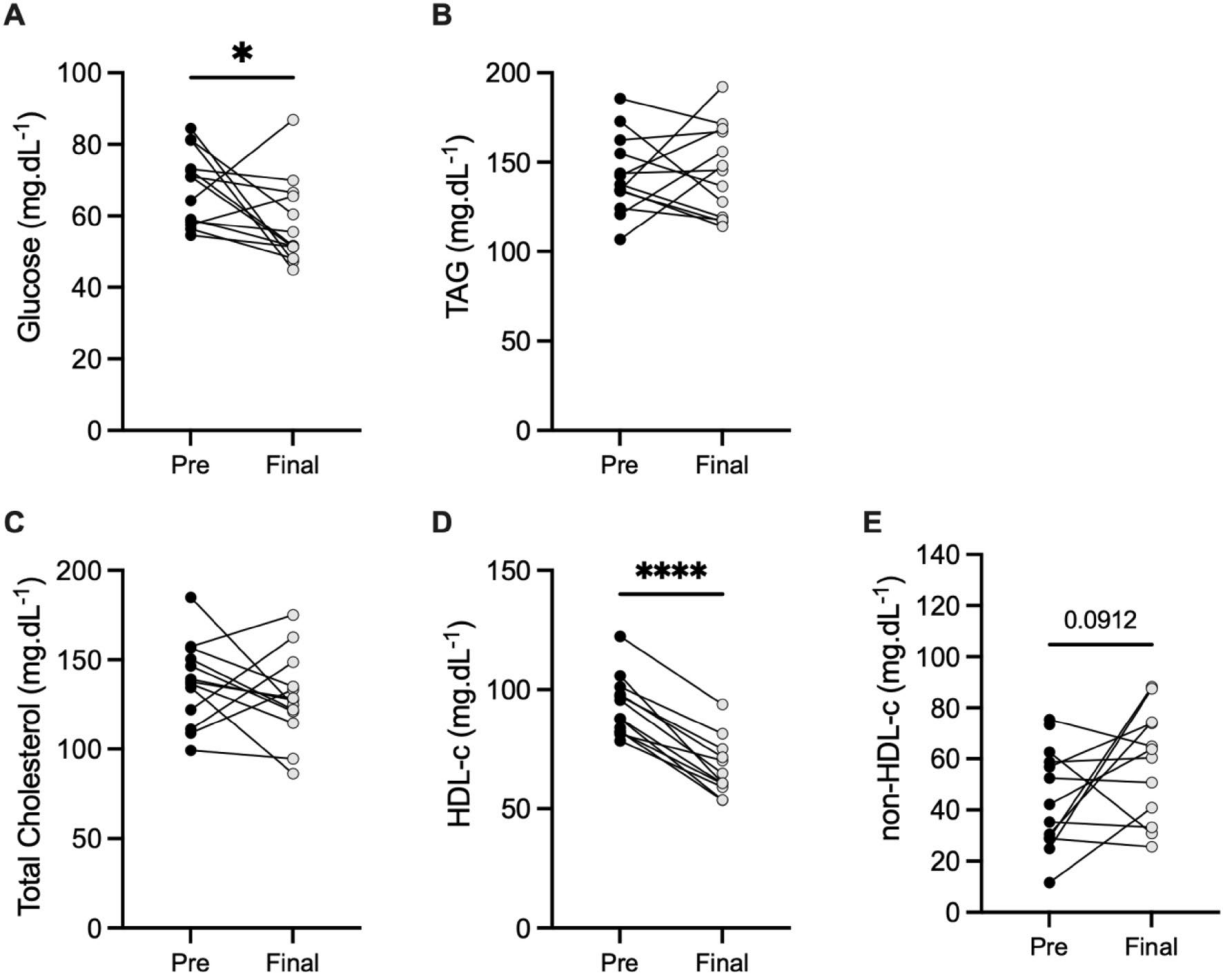
Resting metabolic values before and after badminton season for all individual participants. Individual data points (dots) for the total participants cohort (n = 13) (**A**) glucose, (**B**) triacylglycerol, (**C**) total cholesterol, (**D**) HDL-cholesterol (n = 12), (**E**) non-HDL-cholesterol (n = 12). *P-values indicate differences relative to pre-season resting values.

The first purpose of this study was to examine the metabolic and inflammatory profiles of the athletes at rest, comparing the two chosen seasonal phases. Figure 1 shows the differences of resting values for metabolic parameters and Supplementary table 2 presents the mean and standard deviation of raw data for metabolic and inflammatory markers before and after badminton season. The concentrations of non-fasted glucose (–7.58 [–20.40 to –0.26] mg/dL; p = 0.045; Fig. 1A) and HDL-cholesterol (–26.87 [–31.69 to –20.79] mg/dL; p < 0.0001; Fig. 1D) decreased at the end of the season. Athletes showed a tendency to increased non-HDL-c concentration (9.30 [–2.92 to 33.86] mg/dL; p = 0.091; Fig. 1E) in the final-season, when compared to pre-season. No changes were observed for TAG and total cholesterol (Fig. 1B,C). Badminton athletes exhibited a high interindividual variability in the resting serum cytokine levels between the seasonal points in time. No differences in IL-6, adiponectin and leptin (metabolic), IL-1Ra and IL-10 (anti-inflammatory), TNF-α, IL-17A and IFN-γ (inflammatory), MCP-1 and MIP-1 (migratory) levels were observed in all participants (Suppl. Figure 1). No changes were found in metabolic, pro-inflammatory and anti-inflammatory ratios at rest (Suppl. Figure 2).

### Exercise-response differences between seasonal phases

The second purpose of this study was to compare the metabolic and cytokine response to maximal exercise test between the two seasonal phases. In order to do this, the absolute and fold changes from pre- to post-exercise measurements for metabolic and inflammatory parameters were calculated at beginning and at the end of the season.

**Figure 2:**
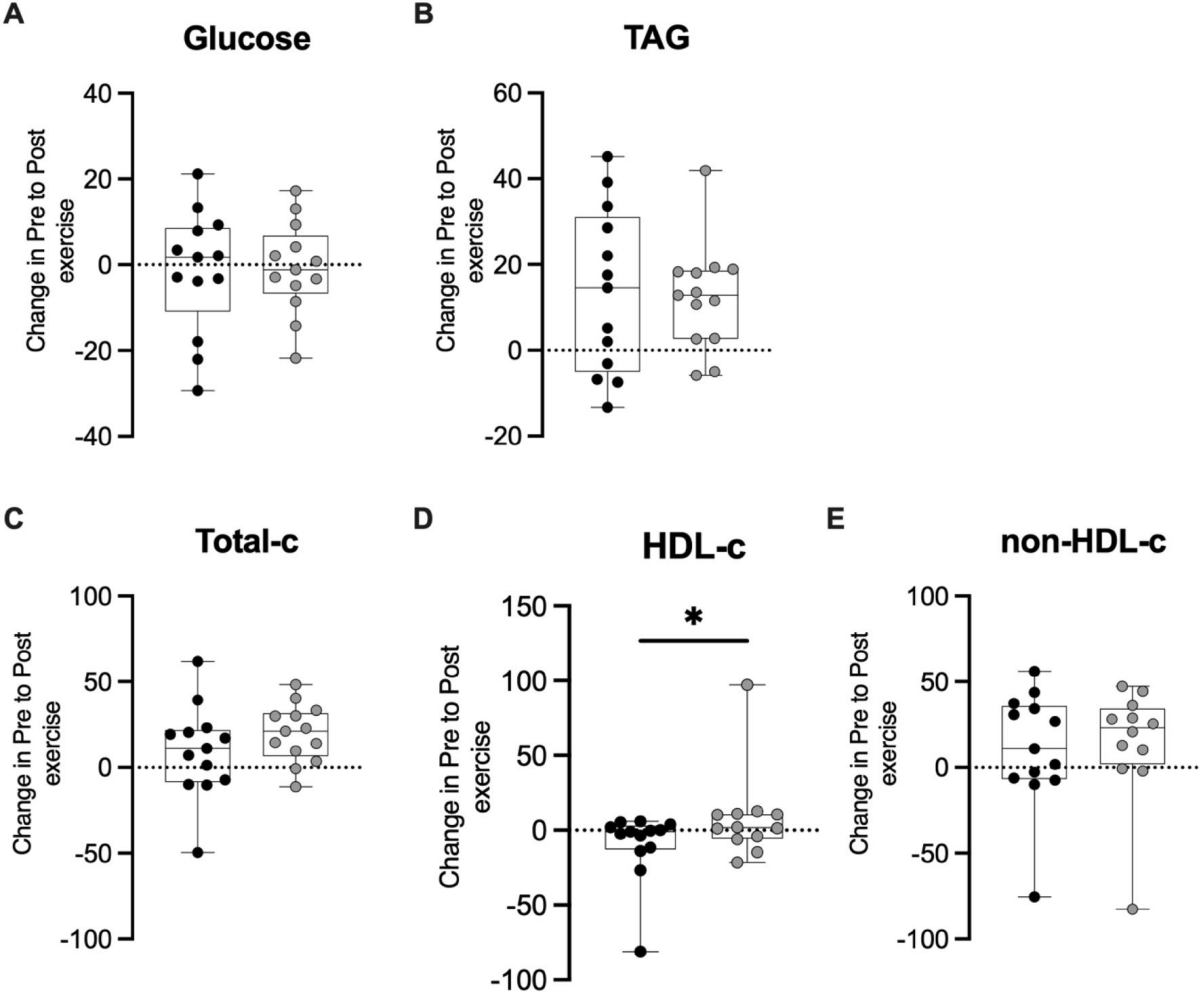
Delta change in metabolic parameters relative to the resting value obtained before and after badminton season. Individual data points (dots) for the total participants cohort (n = 13) (**A**) glucose, (**B**) triacylglycerol, (**C**) total cholesterol, (**D**) HDL-cholesterol, (**E**) non-HDL-cholesterol. *P-values indicate differences relative to pre-season.

There was a significant higher HDL-c variation in response to the maximal test at final-season in comparison to pre-season (+ 10.20 [–1.53 to 39.42] mg/dl; p = 0.042; Fig. 2) as illustrated in Fig. 2. No differences in the delta and fold changes for other metabolic parameters were observed between pre- and post-exercise for both seasonal phases (Fig. 2; Suppl. Figure 3).

**Figure 3:**
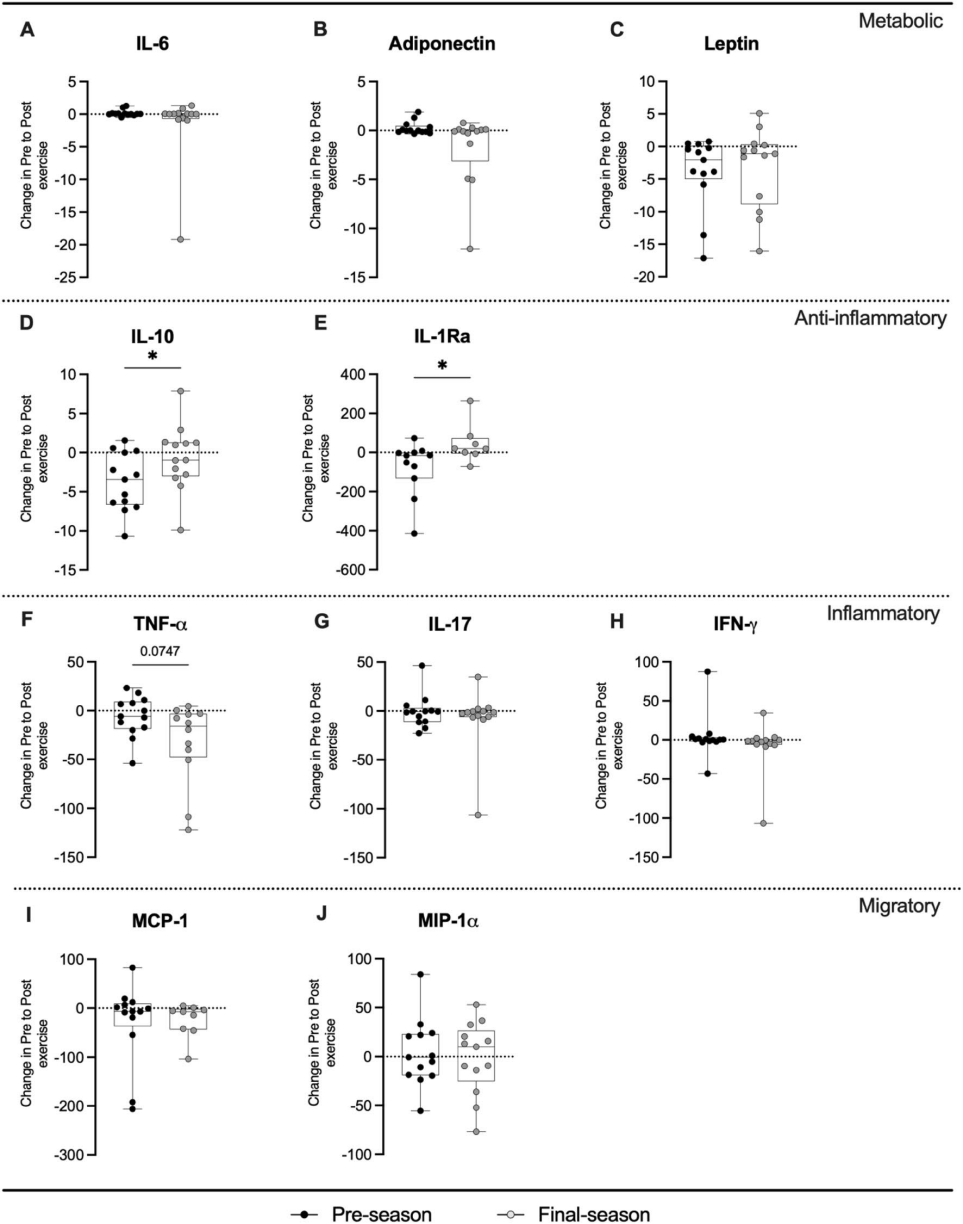
Delta change in cytokine levels relative to the resting value obtained before and after badminton season. Individual data points (dots) for IL-6 (n = 13; **A**), adiponectin (n = 13; **B**), leptin (n = 13; **C**), IL-10 (n = 13; **D**), IL-1ra (n = 6; **E**), TNF-α (n = 12; **F**), IL-17 (n = 13; **G**), IFN-γ (n = 13; **H**), MCP-1 (n = 9; **I**), MIP-1α (n = 13; **J**). For display purposes one participant with high variable fold change values is not included in (**A**) (pre-season: 0.00 pg/ml, final-season: –19.18 pg/ml). *P-values indicate differences relative to the pre-season.

**Figure 4:**
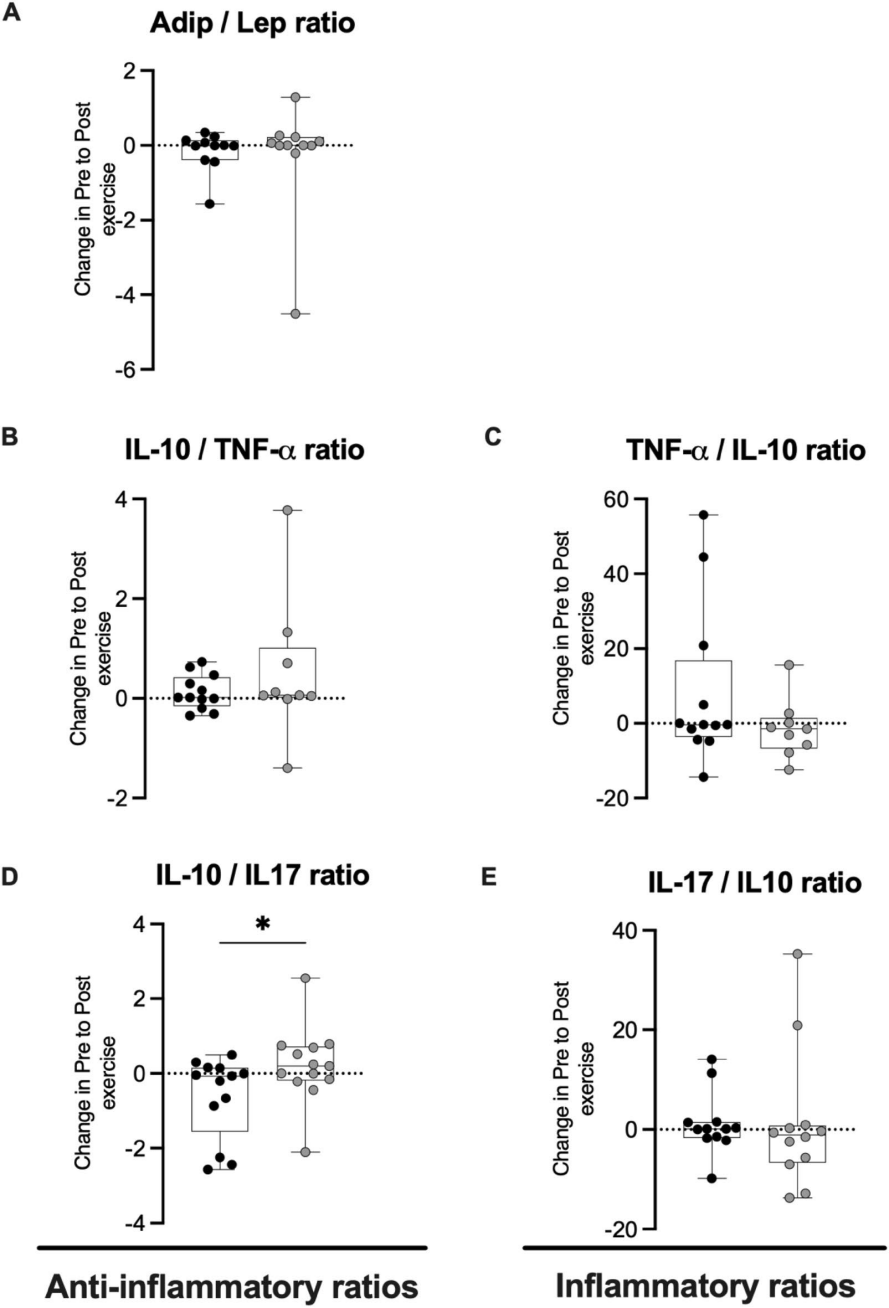
Delta change in metabolic and inflammatory ratios relative to the resting value obtained before and after badminton season. Individual data points (dots) for Adip/Lep ratio (n = 11; **A**), IL-10/TNF-α ratio (n = 10; **B**), IL-10/IL-17 ratio (n = 13; **C**); TNF-α/IL-10 ratio (n = 10; **D**), IL-17/IL-10 ratio (n = 13; **E**). For display purposes one participant with high values is not included in (**A**) (pre-season: 11.67, final-season: –1.13); (**B**) (pre-season: –0.18, final-season: –8.89), (**C**) (pre-season: 3.58, final-season: –388.84), (**E**) (pre-season: 158.3, final-season: 20.41). *P-values indicate differences relative to the pre-season.

Figure 3 shows the differences in cytokine levels at rest between the two seasonal time points. The absolute variation of IL-10 in response to the maximal exercise was greater at the end of the annual badminton season (+ 3.41 [0.31 to 5.89] pg/ml; p = 0.047; Fig. 3D). We observed a 1.4-fold yet IL-10 increase at final-season compared to 0.7-fold change at pre-season (p = 0.066; Suppl. Figure 5). This result was supported by a significant and positive relationship between delta IL-10 and delta VO_2max_ (*r* = 0.62, *p* = 0.03). A similar response was observed for IL-1Ra, with a larger delta change at final season (+ 141.3 [27.35 to 314.0] pg/ml; p = 0.031; Fig. 3E), representing a 2.9-fold increase (p = 0.093; Suppl. Figure 4). The opposite was observed for TNF-α variation, as the TNF-α delta change seemed to be lower at the end of the season (–24.38 [–61.27 to 3.40] pg/ml; p = 0.081; Suppl. 3F). No differences in IL-6, adiponectin, leptin, IL-17, IFN-γ, MCP-1 and MIP-1α were observed in relation to the resting value obtained on pre- and final-season (Fig. 3). The increased exercise-induced IL-10 variation associated with the maintenance of  IL-17 delta change, revealed a significant greater delta change in the IL-10/IL-17 ratio at final-season (+ 0.37 [0.11 to 1.54]; p = 0.010; Fig. 4). No differences were observed in the other metabolic and pro-inflammatory ratios (Fig. 4; Suppl. Figure 5).

## Discussion

The results of this study show that metabolic responses are altered at the end of an annual badminton season in youth athletes, as evidenced by the reduction in the non-fasting glucose and HDL-c values at the final-season when compared to the pre-season measurement. No differences were found in the resting circulating levels of pro- and anti-inflammatory cytokines. However, IL-10 and IL-1Ra response to a maximal intermittent exercise was higher at the end of the season. A trend towards a reduction in TNF-α was also found. We speculate a better synchronism of the immunometabolic input by the scheduled exercise training, as well as an adequate recovery period at the end of the season may explain these fluctuations. Keeping these points in mind, the most recent and relevant literature concerning the outcomes of the present study is discussed below.

In the current study, badminton athletes showed higher fasting HDL-c concentrations (near to 90 mg·dL^−1^). HDL-c plays a critical role in the reverse cholesterol transport process—removing cholesterol from circulation to further transport to the liver for recycling and disposal^17,18^. Thus, higher HDL-c values may reflect an increase in lipid transfer in athletes^19^. Nassef and colleagues conducted an extensive association analysis of aerobic and badminton exercise on HDL-c levels in a large sample size of Taiwanese adults^20^. The participants were categorized based on their exercise status—no exercise (n = 8345), aerobic exercise (n = 4111), and badminton training (n = 49). The authors founded a positive association between HDL-c values and both exercise modalities, with the badminton group being more significant^20^. As far as the association of badminton with HDL-C is concerned, the possible mechanisms underlying these associations remain to be clarified. Most importantly, improvements in HDL function following exercise training are often found in the absence of significant changes in HDL-C levels^21^.

It is worth nothing that badminton athletes showed a significant reduction in fasting HDL-c levels in the final-season compared to pre-season in the present study (− 26.24 ± 8.57 mg/dl; Fig. 1). Nevertheless, the exercise-induced HDL-c variation in badminton athletes was greater in the final-season. Thus, we believe that the modulation in HDL-c values at the end of the season are an adaptative training response, suggesting an efficiency in lipid-related mechanisms (such as the cholesterol efflux), enhanced by regular exercise training. Together with a better efficiency in glycaemic homeostasis observed in these athletes, it seems reasonable to suggest that such metabolic efficiency should have a positive impact upon training status and effectiveness. Thus, these markers could be an additional tool to support routine testing techniques for proper training management by coaches. Further studies are needed to prove this hypothesis.

A preceding study conducted by our research team revealed a reduction in the total calorie and carbohydrate intake by badminton athletes over the season^3^. In the present study, a reduction in resting glucose values was observed at the end of the season, which can be interpreted in different ways. By reducing carbohydrate intake; the level of blood glucose may drop in these athletes. Statically, we did not find any association (r2 = 0.029, P = 0.404) between carbohydrate intake and blood glucose levels. Moreover, the reduction in blood glucose concentration could be due to energy demand induced by strenuous and long-duration exercise^22^, supported by the fact that all participants ate the same breakfast at two seasonal points. More than diet habits, the lower glucose likely could be the result of the taper in training volume^23,24^.

During the annual season the athletes are exposed to exhaustive training loads, schedules with successive games that demand high cognitive and physiological performance and consequently insufficient recovery periods, which may increase the risk of overreaching. Ziemman et al. observed that TNF-α concentration remains elevated after the competitive season among high professional tennis players^25^, reflecting a sustained overreaching, which could have resulted from insufficient recovery after the competitive season. In our study, the badminton athletes decreased serum BDNF levels, sleep time and their total physical activity along the season, the total vigorous activity time and VO_2max_ were similar between pre- and final-season^3^. Thus, we can infer that this group of badminton athletes did not experience overtraining.

In addition, it’s important to mention that athletes can experience short-term performance decrement, without severe psychological, or other lasting negative symptoms like altered inflammatory markers. The data presented herein reveal that the annual badminton season had no impact on resting TNF-α level—a marker of inflammation—corroborating previous results observed in tennis players where TNF-α was unchanged^6,26^. Interestingly, the variation pre- to post-exercise in TNF-α levels was 1.0-fold in pre-season and only 0.5-fold at final-season. Zurek and co-authors showed that TNF-α levels were decreased in highly ranked Polish tennis players towards the end of a preparative training period while controlling workload, diet, and sleep during programmed camps^27^. Because tennis players are not exposed to the same stresses as badminton players, it is conceivable that repeated and transient rises in TNF-α concentration, as a result of each acute exercise session in the badminton training program, mediates a training-induced equillibrium (and maybe a balance) in TNF-α values. These findings let us suggest that the balance between exercise and rest in a competitive badminton season could effectively ameliorate the acute inflammatory response in athletes.

Nonetheless, it may be possible that TNF-α failed to increase due to the improved IL-10 and IL1-Ra response at final-season, which antagonizes the secretion of TNF-α^26^. Anti-inflammatory cytokines such as IL-10 and IL-1Ra plays an important role in orchestrating the inflammatory response, inhibiting pro-inflammatory activities of various cytokines. Herein, the mean concentration of the two cytokines remained unchanged at resting condition or after acute-exercise in badminton athletes, regardless of the season time-points studied. Interestingly, our results revealed that exercise-induced IL-10 and IL-1Ra variation are different between pre- and final-season. Hence, one may speculate that the largest IL-10 and IL-1Ra variation in response to the Yo-Yo test could reflets more responsivity of these cytokines to the maximal exercise at this time point in badminton athletes. The mechanisms underlying this difference remain to be clarified.

While IL-10 is a cytokine with a potent anti-inflammatory properties, IL-17 is the signature pro-inflammatory cytokine secreted by Th17 cells, and might reflect muscle damage^28^. Hacker et al. investigated a larger number of blood-based biomarkers and their regulation with regard to different recovery states in male badminton and soccer athletes^5^. A recovered state was attributed to a minimum one day of rest after a habitual loading microcycle, while the analyses of biomarkers after the last training session was the non-recovered condition in athletes. The authors founded an elevated IL-17A concentrations in athletes at the non-recovered compared to recovered condition^5^. Here, no change in IL-17 values was observed at baseline and after to the maximal exercise test, contradicting our initial hypothesis that these athletes would show an elevation in the immune-inflammatory and metabolic responses, as an “residual effect” in relation to the training load and demands on the sport in the annual season.

Furthermore, in training and competition conditions, it is important to maintain a balance between pro- and anti-inflammatory responses. Keeping this in view, inflammatory ratios are strong indicators to appropriately reflect habitual inflammatory status. In addition, adiponectin/leptin ratio is an important parameter that reflects the functionality of the adipose tissue and it was speculated that the rise in Adip/Lep ratio could be a compensatory response to systemic inflammation. In the present study, a greater exercise-response in IL-10/IL-17 ratio was reported at final-season, while no differences in the Adip/Lep and other metabolic and pro-inflammatory ratios were observed. We speculate that favourable metabolic environment and possible improvement in anti-inflammatory response observed in the present study, may be the reason why we found no changes in this setting. Otherwise, the ability of young athletes to respond the inflammatory stimulus given by acute exercise even at the end of an entire season, could maintain an “inflammatory balance”, avoiding the physiological and metabolic consequences of the pro-inflammatory status.

In the present study, we provide evidence that an entire season of badminton training impacts the immunometabolic responses; however, metabolic and inflammatory markers were changed differently in two contrasting—early and late—season phases in youth badminton athletes. Although fasting metabolic parameters are influenced by badminton season, the inflammatory challenge imposed by maximal exercise seems to be more important than resting values to verify the actual effect of a training season in badminton athletes, being a relevant piece of information in terms of immune adaptation. For the first time our results showed an elevated IL-10 and IL-1Ra variation in response to a maximal exercise even at the end of an entire season. The major responsivity of these cytokines at this time point suggests the ability of young badminton athletes to respond the inflammatory stimulus given by acute exercise. Herein, we can speculate that such time window improved the immunometabolic efficiency of these athletes and that they became more effective in their substrate expenditure and acute inflammatory response. Such efficiency should have a positive impact upon training effectiveness and maintenance of the cardio-metabolic health of the athletes. Accordingly, a sport-practical relevant advice on training management is that at the end of a season, compared to the beginning of a season, the same load is much more strenuous and more inflammatory. In conclusion, coaches should reduce the training load at the end of the season in order to reduce or avoid high inflammatory processes in badminton players, also looking to interindividual variability of these markers. Therefore, the results of this study may be applied by coaches, trainers and sport nutritionist for proper training management.

Despite the importance of the data here presented, some considerations should be mentioned. For example, we only measured the metabolic and inflammatory parameters at two time-points (pre- vs. final-season) during the entire season. As such, increasing the number of measurements should be considered in the future studies to improve temporal profiling. While the small number of participants in this study could be considered a limitation, especially to verify the potential difference between season time-points, its reflects an entire badminton time which mitigates this drawback. The menstrual cycle was not tracked in the female athletes. Furthermore, comparisons by genders and other recovery-stress biomarkers, such as hormones and physiological factors should be considered in future studies.

## Supplementary Information

Supplementary Information.

## Data Availability

The data that support the findings of this study are available from the corresponding author upon request.

## References

[1] Phomsoupha M, Laffaye G (2015). The science of badminton: Game characteristics, anthropometry, physiology, visual fitness and biomechanics. Sports Med..

[2] Kellmann M (2010). Preventing overtraining in athletes in high-intensity sports and stress/recovery monitoring. Scand. J. Med. Sci. Sports.

[3] Santos AMS (2021). Brain-derived neurotrophic factor and psychophysiological response in youth badminton athletes during the season. Int. J. Sports Physiol. Perform..

[4] Padilha CS (2021). Immunometabolic responses according to physical fitness status and lifelong exercise during aging: New roads for exercise immunology. Ageing Res. Rev..

[5] Hacker S (2021). Recovery-stress response of blood-based biomarkers. Int. J. Environ. Res. Public Health.

[6] Witek K (2016). Myokines in response to a tournament season among young tennis players. Biomed. Res. Int..

[7] Kostrzewa-Nowak D, Nowak R (2020). T helper cell-related changes in peripheral blood induced by progressive effort among soccer players. PLoS ONE.

[8] Lee EC (2017). Biomarkers in sports and exercise: Tracking health, performance, and recovery in athletes. J. Strength Cond. Res..

[9] Ortega E (2016). The, “bioregulatory effect of exercise” on the innate/inflammatory responses. J. Physiol. Biochem..

[10] Petersen AMW (2005). The anti-inflammatory effect of exercise. J. Appl. Physiol..

[11] Ostrowski K, Rohde T, Asp S, Schjerling P, Pedersen BK (1999). Pro- and anti-inflammatory cytokine balance in strenuous exercise in humans. J. Physiol..

[12] Tilg H, Trehu E, Atkins MB, Dinarello CA, Mier JW (1994). Interleukin-6 (IL-6) as an anti-inflammatory cytokine: Induction of circulating IL-1 receptor antagonist and soluble tumor necrosis factor receptor p55. Blood.

[13] Ho CS, Lee MC, Chang CY, Chen WC, Huang WC (2020). Beneficial effects of a negative ion patch on eccentric exercise-induced muscle damage, inflammation, and exercise performance in badminton athletes. Chin. J. Physiol..

[14] Lichtenstein AH (2006). Diet and lifestyle recommendations revision 2006: A scientific statement from the American heart association nutrition committee. Circulation.

[15] Bangsbo J, Iaia FM, Krustrup P (2008). The Yo-Yo intermittent recovery test. Sport. Med..

[16] Léger LA, Mercier D, Gadoury C, Lambert J (1988). The multistage 20 metre shuttle run test for aerobic fitness. J. Sports Sci..

[17] Camont L, Chapman MJ, Kontush A (2011). Biological activities of HDL subpopulations and their relevance to cardiovascular disease. Trends Mol. Med..

[18] Marques LR (2018). Reverse cholesterol transport: Molecular mechanisms and the non-medical approach to enhance HDL cholesterol. Front. Physiol..

[19] Vaisberg M (2012). Lipid transfer to HDL is higher in marathon runners than in sedentary subjects, but is acutely inhibited during the run. Lipids.

[20] Nassef Y (2019). The impact of aerobic exercise and badminton on HDL cholesterol levels in adult Taiwanese. Nutrients.

[21] Ruiz-Ramie JJ, Barber JL, Sarzynski MA (2019). Effects of exercise on HDL functionality. Curr. Opin. Lipidol..

[22] Von Ah Morano AE, Dorneles GP, Peres A, Lira FS (2020). The role of glucose homeostasis on immune function in response to exercise: The impact of low or higher energetic conditions. J. Cell. Physiol..

[23] Rossi FE, de Freitas MC, Zanchi NE, Lira FS, Cholewa JM (2018). The role of inflammation and immune cells in blood flow restriction training adaptation: A review. Front. Physiol..

[24] de Freitas MC, Gerosa-Neto J, Zanchi NE, Lira FS, Rossi FE (2017). Role of metabolic stress for enhancing muscle adaptations: Practical applications. World J. Methodol..

[25] Ziemann E (2012). Five-day whole-body cryostimulation, blood inflammatory markers, and performance in high-ranking professional tennis players. J. Athl. Train..

[26] Kozłowska M (2021). Immunological response and match performance of professional tennis players of different age groups during a competitive season. J. Strength Cond. Res..

[27] Żurek P (2022). Planned physical workload in young tennis players induces changes in iron indicator levels but does not cause overreaching. Int. J. Environ. Res. Public Health.

[28] Sugama K, Suzuki K, Yoshitani K, Shiraishi K, Kometani T (2012). IL-17, neutrophil activation and muscle damage following endurance exercise. Exerc. Immunol. Rev..

