# Supplementary material for: Exercise training-induced changes in immunometabolic markers in youth badminton athletes
Source: Sci Rep. 2022 Sep 15;12:15539. doi: 10.1038/s41598-022-19591-w (PMC9477844; doi:10.1038/s41598-022-19591-w)
Supplement: Supplementary file 1 — Supplementary Information. [file 41598_2022_19591_MOESM1_ESM.docx]

**Supplementary material**

**Suppl. Table 1.** The intra- and inter-assay variations (%) and the sensitivity of the enzymatic kit.

|  | Sensitivity (pg/mL) | Intra-assay (CV %) | Inter-assay (CV %) |
| --- | --- | --- | --- |
| IL-6 | 0.7 | 2.6 | 4.5 |
| TNF-α | 6.2 | 4.6 | 5.8 |
| IL-10 | 3.9 | 3.6 | 6.9 |
| IL-1ra | - | 4.0 | 8.7 |
| Leptin | - | 7.6 | 9.7 |
| Adiponectin | - | 2.8 | 2.4 |
| IL-17 | - | 7.6 | 9.7 |
| MCP-1 | - | 9.2 | 9.1 |
| MIP-1α | - | 6.8 | 9.8 |

**Suppl. Table 2.** Mean(SD) of raw data for metabolic and inflammatory markers before and after badminton season.

|  | Pre-season | | | | Final-season | | | |
| --- | --- | --- | --- | --- | --- | --- | --- | --- |
|  | Pre | | Post | | Pre | | Post | |
|  | N | Mean(SD) | N | Mean(SD) | N | Mean(SD) | N | Mean(SD) |
| **Cytokines** |  |  |  |  |  |  |  |  |
| IL-6 (pg/ml) | 13 | 3.0(0.2) | 13 | 3.2(0.5) | 13 | 4.8(5.4) | 13 | 3.3(0.7) |
| Adiponectin (ug/ml) | 12 | 5.7(8.0) | 13 | 5.5(7.8) | 13 | 5.7(7.7) | 12 | 4.3(7.2) |
| Leptin (ng/ml) | 13 | 13.2(10.8) | 13 | 9.4(9.1) | 13 | 13.2(10.8) | 13 | 9.4(9.1) |
| IL-10 (pg/ml) | 13 | 7.9(5.2) | 13 | 4.2(3.1) | 13 | 5.1(3.4) | 13 | 4.4(3.9) |
| IL-1Ra (pg/ml) | 12 | 364.4(792.5) | 11 | 316.9(861.8) | 9 | 308.4(665.7) | 9 | 380.9(744.3) |
| TNF-α (pg/ml) | 12 | 29.7(13.9) | 12 | 25.6(17.4) | 12 | 46.4(54.2) | 10 | 16.1(20.5) |
| IL-17A (pg/ml) | 13 | 69.2(170.1) | 13 | 68.6(184.3) | 13 | 76.1(173.9) | 13 | 68.6(184.5) |
| IFN-γ (pg/ml) | 13 | 39.7(90.1) | 13 | 43.8(94.1) | 13 | 36.2(82.2) | 11 | 43.2(91.4) |
| MCP-1 (pg/ml) | 13 | 103.9(189.3) | 13 | 75.3(189.6) | 10 | 58.8(80.9) | 10 | 37.2(76.3) |
| MIP-1α (pg/ml) | 13 | 191.0(68.6) | 13 | 194.9(65.5) | 13 | 182.8(53.0) | 13 | 181.6(58.0) |
|  |  |  |  |  |  |  |  |  |
| **Metabolic profile** |  |  |  |  |  |  |  |  |
| Glucose (mg·dL^-1^) | 13 | 68.1(10.4) | 13 | 66.5(9.0) | 13 | 57.7(11.8) | 13 | 56.9(10.2) |
| TAG (mg·dL^-1^) | 13 | 142.7(21.7) | 13 | 156.3(30.3) | 13 | 144.7(25.2) | 13 | 157.0(26.2) |
| TC (mg·dL^-1^) | 13 | 137.4(23.2) | 13 | 146.9(23.9) | 13 | 128.8(24.2) | 13 | 148.4(26.1) |
| HDL-c (mg·dL^-1^) | 12 | 93.5(12.5) | 12 | 90.0(15.5) | 12 | 67.1(11.9) | 12 | 75.4(28.6) |
| Non-HDL-c (mg·dL^-1^) | 12 | 42.2(18.5) | 12 | 60.2(20.4) | 12 | 57.9(21.6) | 12 | 71.7(36.3) |
|  |  |  |  |  |  |  |  |  |
| **Ratios** |  |  |  |  |  |  |  |  |
| TNF-α/IL-10 ratio | 13 | 7.2(9.1) | 13 | 15.1(19.4) | 12 | 46.3(115.7) | 11 | 5.7(5.7) |
| IL-10/TNF-α ratio | 13 | 0.3(0.2) | 13 | 0.3(0.3) | 12 | 1.1(2.5) | 11 | 0.8(1.2) |
| IL-17/IL-10 ratio | 13 | 17.8(49.2) | 13 | 31.1(69.1) | 13 | 14.4(24.7) | 13 | 17.0(27.4) |
| IL-10/IL-17 ratio | 13 | 1.6(1.9) | 13 | 1.0(1.2) | 13 | 0.6(0.7) | 13 | 0.8(0.9) |
| Adip/Lep ratio | 12 | 1.9(3.0) | 12 | 2.7(5.3) | 13 | 1.6(2.5) | 12 | 1.1(2.0) |

**Note**: Values for N below 13 represent undetectable cases in the ELISA measurement.

**
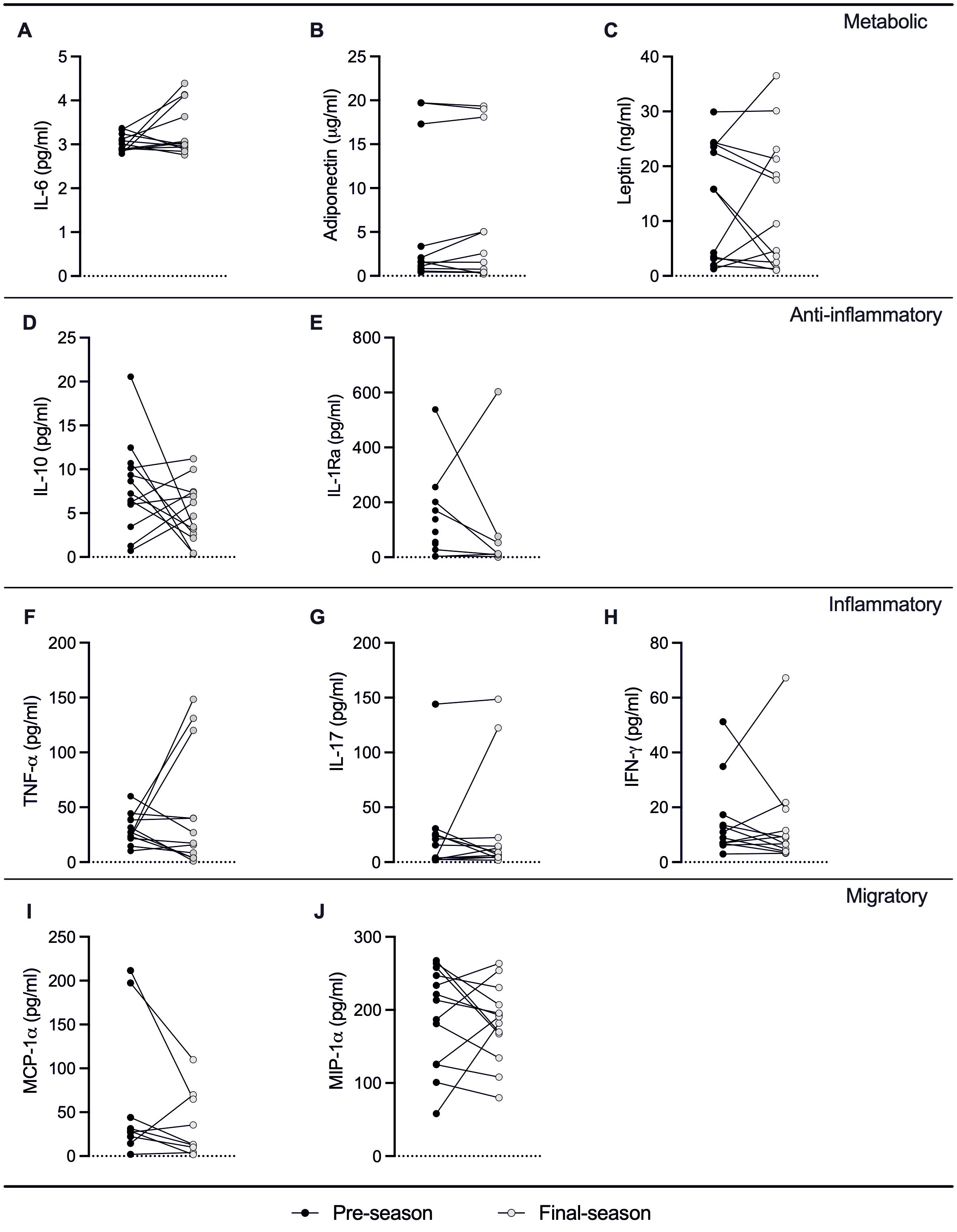
**

**Suppl. Fig 1. Resting serum cytokine levels before and after badminton season for all individual participants.** Individual data points (dots) for IL-6 (n=12; A), Adiponectin (n=12; B), Leptin (n=13; C), IL-10 (n=13; D), IL-1ra (Pre, n=11; Final, n=8; E), TNF-α (n=12; F), IL-17 (n=12; G), IFN-γ (n=12; H), MCP-1 (n=10; I), MIP-1α (n=13; J). For display purposes one participant with high serum cytokine values is not included in panel A (pre-season: 2.77 pg/ml, final-season: 22.90 pg/ml), panel E (pre-season: 2836.01 pg/ml, final-season: 2007.01 pg/ml), panel G panel I (pre-season: 621.06 pg/ml, final-season: 632.51 pg/ml), panel H (pre-season: 336.0 pg/ml, final-season: 303.70 pg/ml), panel I (pre-season: 691.05 pg/ml, final-season: 265.70 pg/ml). * P-values indicate differences relative to the pre-season resting values.

**
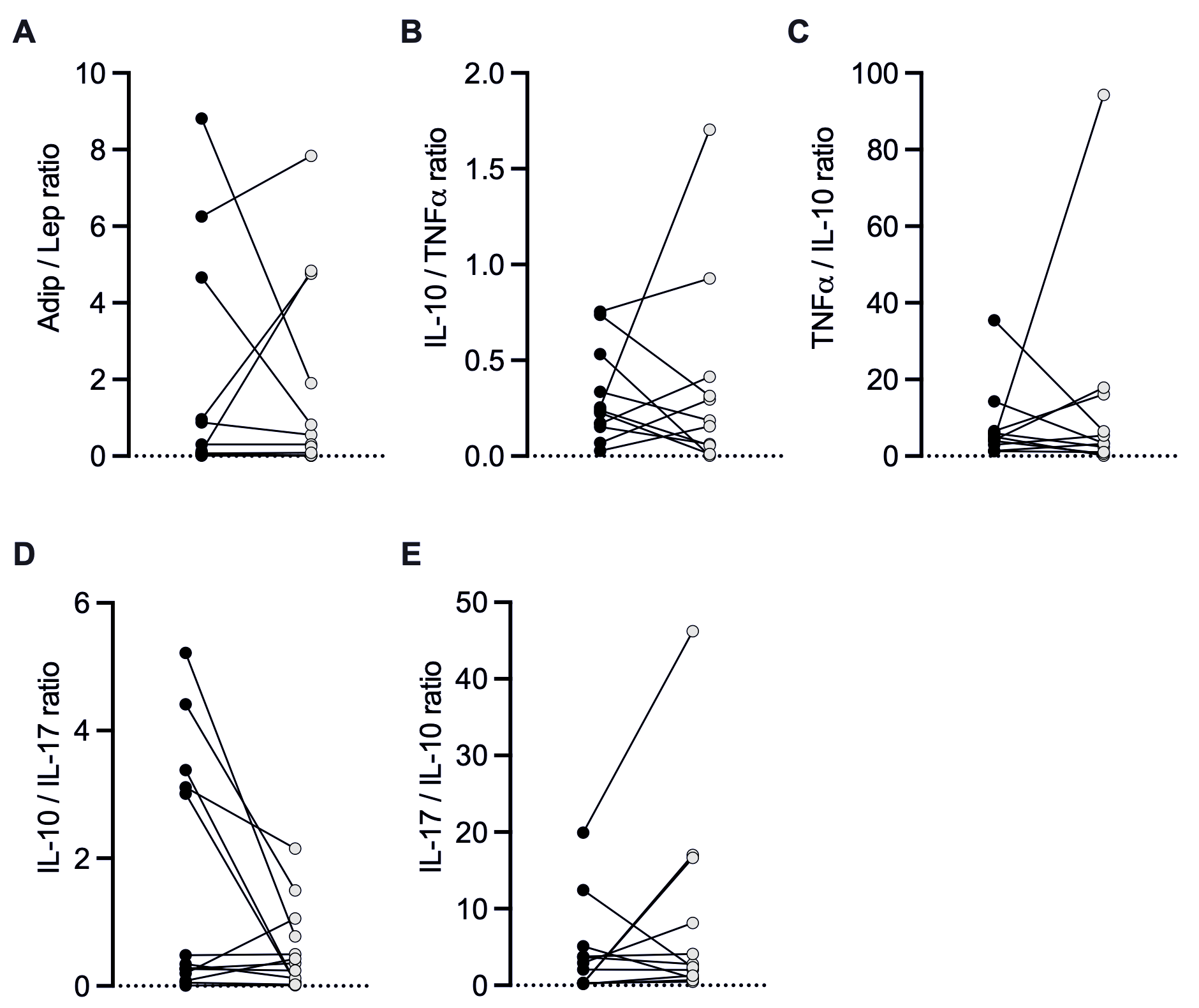
**

**Suppl. Fig 2. Resting metabolic and inflammatory ratios values before and after badminton season for all individual participants.** Individual data points (dots) for Adip/Lep ratio (n=12; A), IL-10/TNF-α ratio (n=12; B), IL-10/IL-17 ratio (n=13; C); TNF-α/IL-10 ratio (n=12; D), IL-17/IL-10 (n=13; E). For display purposes one participant with high values is not included in panel B (pre-season: 0.19, final-season: 8.95); panel C (pre-season: 1.88, final-season: 303.7), panel E (pre-season: 180.38, final-season: 84.85). * P-values indicate differences relative to the pre-season resting values.

**
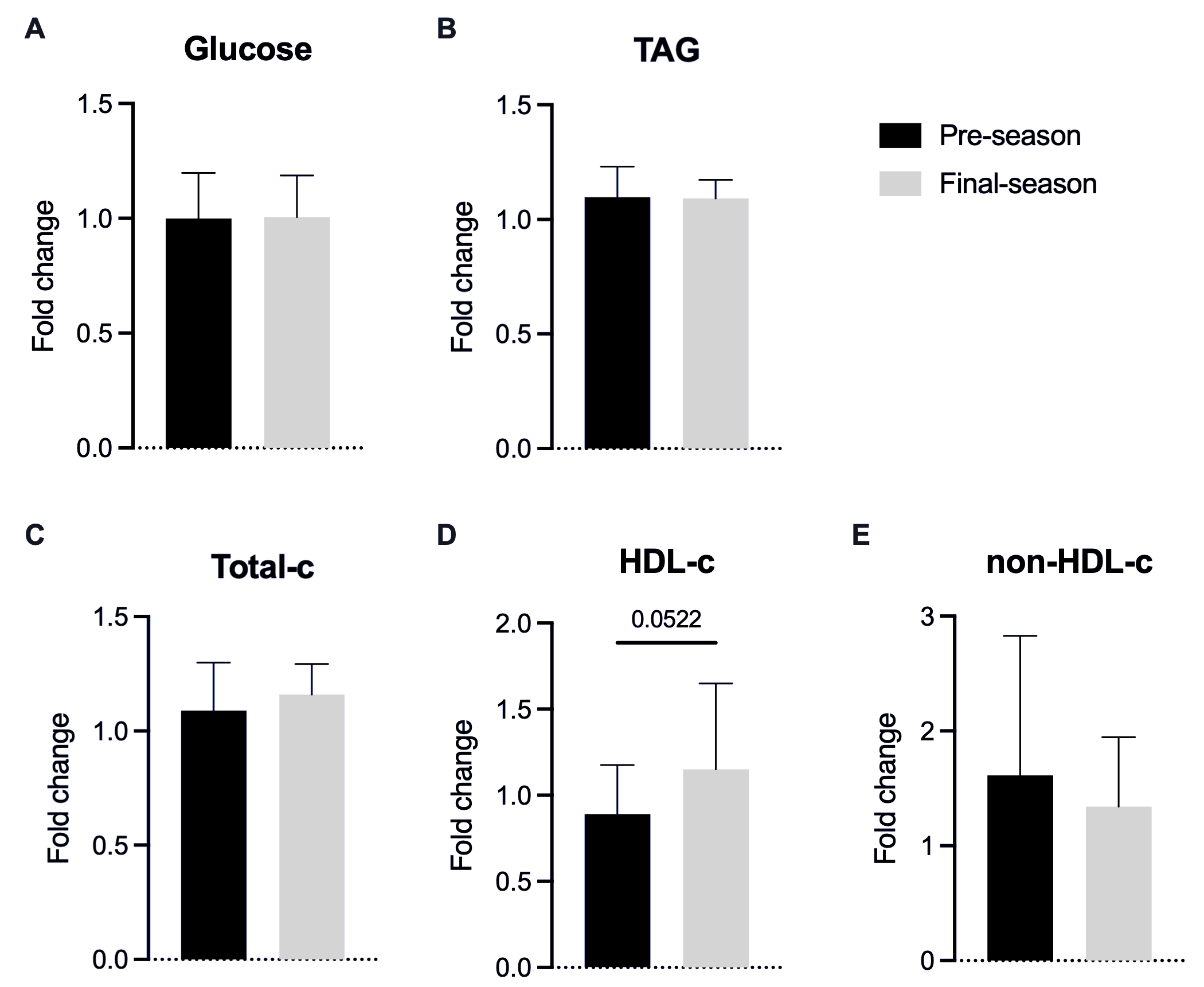
**

**Suppl. Figure 3. Fold change in metabolic parameters relative to the resting value obtained before and after badminton season.** Bar graphs show mean and standard deviation (normalized data) or median and confidence interval (non-normal data) for all participants cohort (n=13) (A) glucose, (B) triacylglycerol, (C) Total Cholesterol, (D) HDL-cholesterol (n=12), and (E) non-HDL-cholesterol (n=12). * P-values indicate differences relative to pre-season.

**
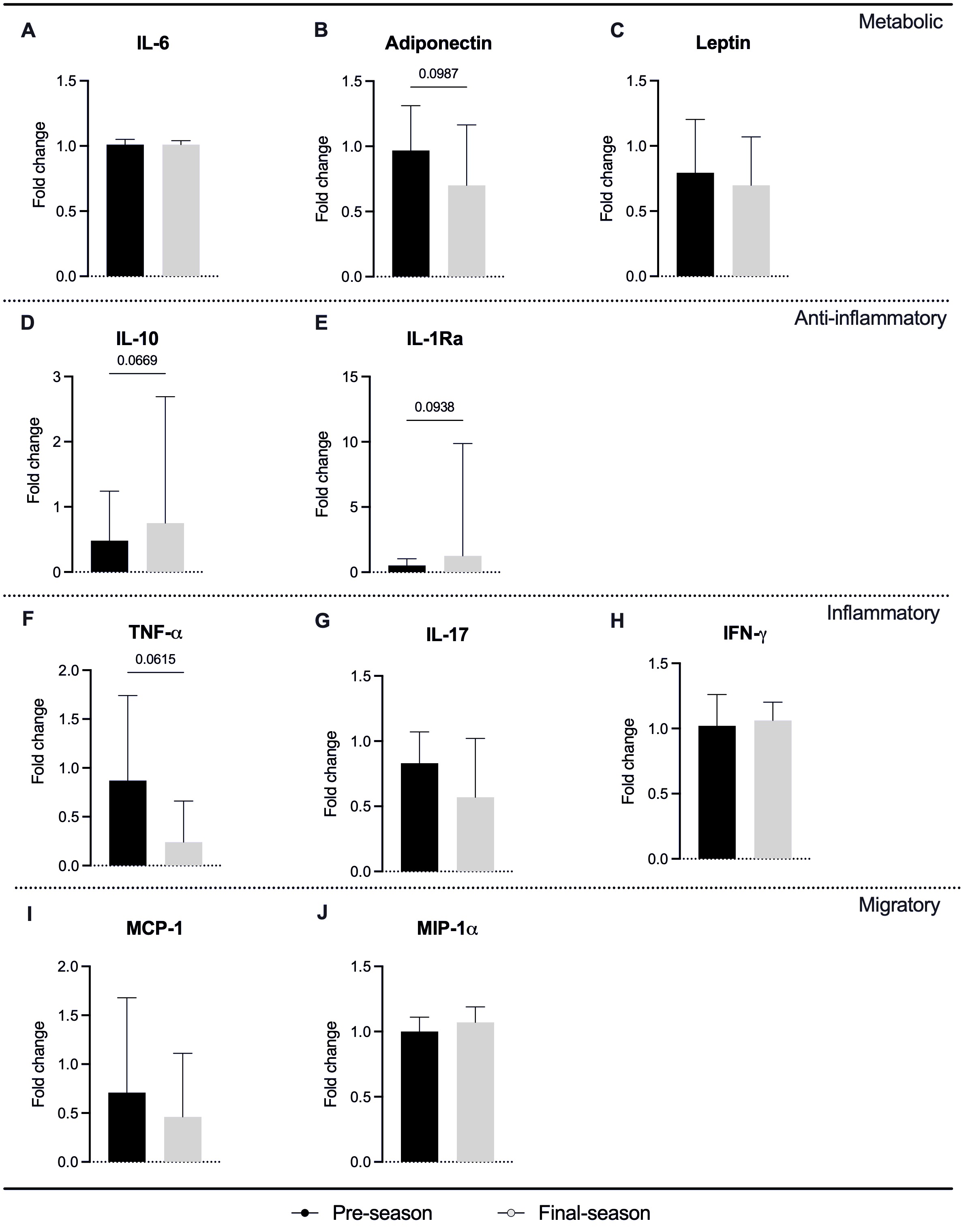
**

**Suppl. Figure 4. Fold change in cytokine levels relative to the resting value obtained before and after badminton season.** Bar graphs show mean and standard deviation (normalized data) or median and confidence interval (non-normal data) for IL-6 (n=13; A), Adiponectin (n=13; B), Leptin (n=13; C), IL-10 (n=13; D), IL-1ra (n=6; E), TNF-α (n=12; F), IL-17 (n=13; G), IFN-γ (n=13; H), MCP-1 (n=9; I), MIP-1α (n=13; J). * P-values indicate differences relative to the pre-season.


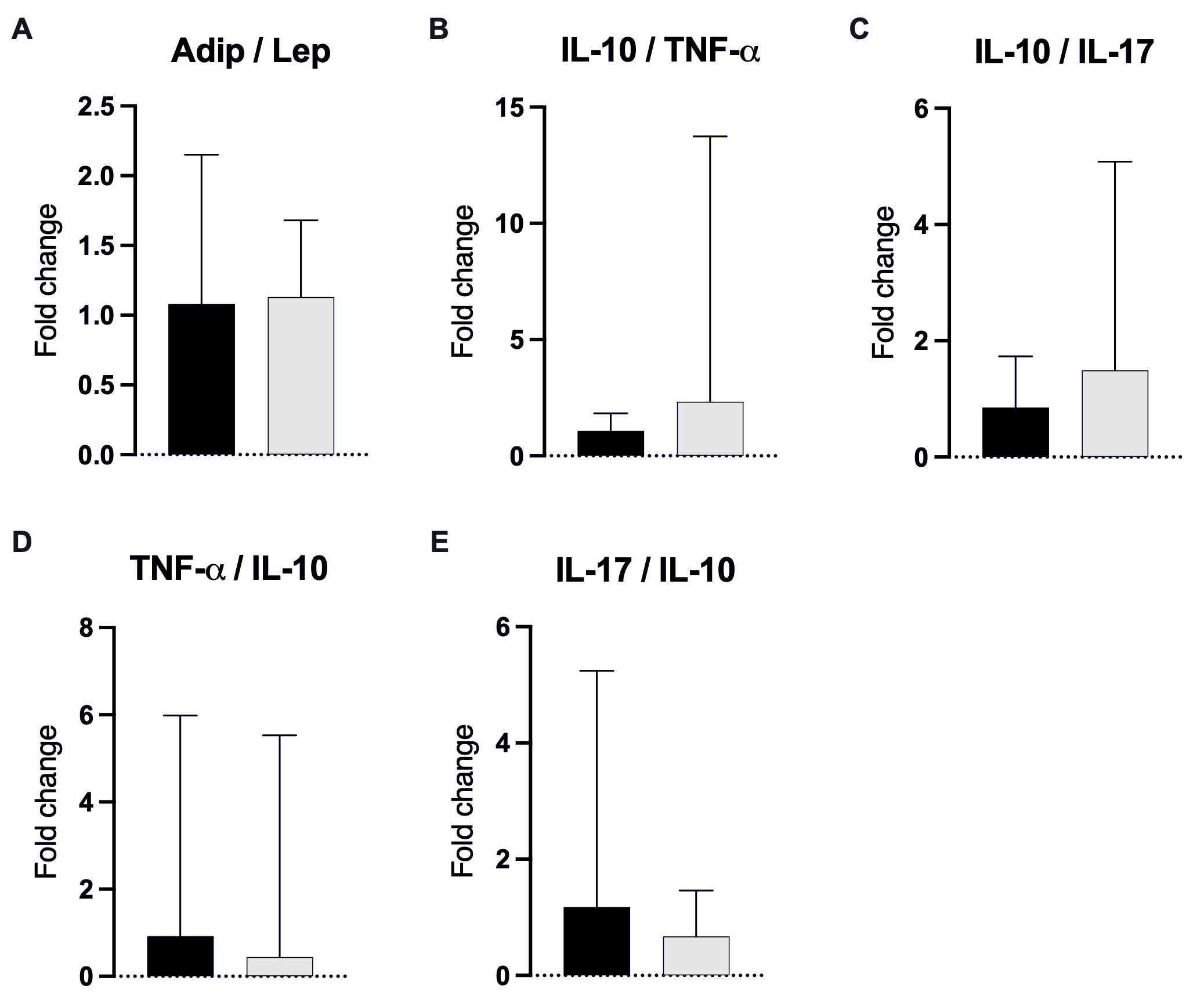


**Suppl. Fig 5. Fold change in metabolic and inflammatory ratios values relative to the resting value obtained before and after badminton season.** Bar graphs show mean and standard deviation (normalized data) or median and confidence interval (non-normal data) for Adip/Lep ratio (n=11; A), IL-10/TNF-α ratio (n=10; B), IL-10/IL-17 ratio (n=13; C); TNF-α/IL-10 ratio (n=10; D), IL-17/IL-10 (n=13; E). * P-values indicate differences relative to the pre-season.
